# Chest wall thickness and depth to vital structures in paediatric patients – implications for prehospital needle decompression of tension pneumothorax

**DOI:** 10.1186/s13049-019-0623-5

**Published:** 2019-04-16

**Authors:** Tom Terboven, Georg Leonhard, Lucas Wessel, Tim Viergutz, Marcus Rudolph, Michael Schöler, Meike Weis, Holger Haubenreisser

**Affiliations:** 1 Department of Anaesthesiology and Intensive Care Medicine, University Medical Center Mannheim, Theodor-Kutzer-Ufer 1-3, 68167 Mannheim, Germany; 2 Department of Paediatric Surgery, Mannheim University Medical Center, Theodor-Kutzer-Ufer 1-3, 68167 Mannheim, Germany; 3 DRF Stiftung Luftrettung gemeinnützige AG, Filderstadt, Germany; 4 Institute of Clinical Radiology and Nuclear Medicine, University Medical Center Mannheim, Theodor-Kutzer-Ufer 1-3, 68167 Mannheim, Germany

**Keywords:** Tension pneumothorax, Needle decompression, Children, Paediatric, Chest wall thickness, Complications

## Abstract

**Background:**

Recommendations regarding decompression of tension pneumothorax in small children are scarce and mainly transferred from the adult literature without existing evidence for the paediatric population. This CT-based study evaluates chest wall thickness, width of the intercostal space (ICS) and risk of injury to vital structures by needle decompression in children.

**Methods:**

Chest wall thickness, width of the intercostal space and depth to vital structures were measured and evaluated at 2nd ICS midclavicular (MCL) line and 4th ICS anterior axillary line (AAL) on both sides of the thorax using computed tomography (CT) in 139 children in three different age groups (0, 5, 10 years).

**Results:**

Width of the intercostal space was significantly smaller at the 4th ICS compared to the 2nd ICS in all age groups on both sides of the thorax. Chest wall thickness was marginally smaller at the 4th ICS compared to the 2nd ICS in infants and significantly smaller at 4th ICS in children aged 5 years and 10 years. Depth to vital structure for correct angle of needle entry was smaller at the 4th ICS in all age groups on both sides of the thorax. Incorrect angle of needle entry however is accompanied by a higher risk of injury at 2nd ICS. Furthermore, in some children aged 0 and 5 years, the heart or the thymus gland were found directly adjacent to the thoracic wall at 2nd ICS midclavicular line.

**Conclusion:**

Especially in small children risk of iatrogenic injury to vital structures by needle decompression is considerably high. The 4th ICS AAL offers a smaller chest wall thickness, but the width of the ICS is smaller and the risk of injury to the intercostal vessels and nerve is greater. Deviations from correct angle of entry however are accompanied by higher risk of injury to intrathoracic structures at the 2nd ICS. Furthermore, we found the heart and the thymus gland to be directly adjacent to the thoracic wall at the 2nd ICS MCL in a few children. From our point of view this puncture site can therefore not be recommended for decompression in small children. We therefore recommend 4th ICS AAL as the primary site of choice.

**Electronic supplementary material:**

The online version of this article (10.1186/s13049-019-0623-5) contains supplementary material, which is available to authorized users.

## Background

Traumatic or spontaneous tension pneumothorax is a potentially fatal event that requires immediate decompression. Currently recommended interventions for decompression are either needle thoracostomy or open finger thoracostomy [1, 2]. Needle thoracostomy is generally easier to learn, faster to perform and less invasive than surgical decompression. It therefore represents the preferred first line technique for many emergency providers and is recommended in several trauma guidelines [2, 3]. However, recent ATLS 10th edition guidelines suggest 4th/5th ICS mid-axillary line as preferable to 2nd ICS in adults and recommends same management for pneumothorax in children, except for needle or tube size. 2nd ICS MCL is still recommended as a possible insertion site in children [4]. There is no reference in the APLS 6th edition on depth of insertion and it recommends 2nd ICS insertion site only [5]. Commonly recommended puncture techniques are insertion sagittal to the chest wall at the 2nd intercostal space (ICS) in the midclavicular line (MCL) and perpendicular to the chest wall at the 4th or 5th ICS anterior axillary line (AAL) or midaxillary line (MAL). However, failed decompression is a commonly reported phenomenon in needle thoracostomy [6]. Therefore, in recent years, several studies examining chest wall thickness (CWT) at the recommended insertion sites have been conducted in adult patients and found commonly used cannulas being too short for successful decompression in a high proportion of patients [6]. This has led to the recommendation of using longer 7-8 cm catheters for needle thoracostomy in adult patients [7–9]. Even though this increases the likelihood of successful decompression, it also increases the risk of injuring underlying vital structures like large intrathoracic vessels or the heart because of the possibility of deeper insertion [9]. Due to the smaller anatomic structures, tension pneumothorax represents a particular challenge in paediatric patients. Open finger thoracostomy in the very narrow intercostal spaces in children requires smaller instruments and some surgical skills, which are often not available in the prehospital setting. Especially in small children it is accompanied by the risk of too large incisions with leakage of air along chest tubes. Furthermore, whilst the technique is similar to an adult, invasive paediatric critical procedures are often associated with cognitive hurdles and dissonance. Little is known about the required insertion depth of a needle for decompression in paediatrics or the likelihood of injuring underlying vital structures. Because of the narrow intercostal space, the risk of injury to the intercostal vessels and nerves has to be taken into consideration when performing needle decompression. We aimed to evaluate the required depth for successful decompression, defined as the distance from skin to pleural space, whilst minimizing iatrogenic underlying structure injury.

Therefore, the primary aim of this study was evaluation of the insertion site recommended by APLS guidelines (2nd ICS MCL) regarding risk of injury to intrathoracic vital structures. Secondary aims were assessing required insertion depth of the needle for successful decompression and measuring width of the intercostal space to study if finger thoracostomy is possible. Furthermore, we investigated the same questions at the 4th ICS AAL as an alternative insertion site.

## Methods

Inclusion criteria were meeting one of the required age groups and availability of a thoracic CT scan in the local picture communication and archiving system. A total of 197 paediatric patients referred for thoracic CT with various indications were initially included in this study. We excluded all patients with a condition that made one or more of the measurements impossible or inaccurate. Consequently 58 patients were excluded due to various pulmonary pathologies which made the required measurements impossible or invalid. Most of the excluded patients were infants with large intrathoracic pathologies or conditions resulting in mediastinal shift. Details on reasons for exclusion are shown in Table 1. The remaining 139 patients were included in three study groups aged 0, 5 and 10 years.

**Table 1:** Reasons for exclusion

Reason for exclusion	n (0 years)	n (5 years)	n (10 years)	n (total)
Mediastinal shift	12	1	1	14
Pulmonary infiltration	11	1	0	12
CPAM	8	0	0	8
Pulmonary bullae	6	0	0	6
Pleural effusion/empyema	3	2	0	5
Poor image quality	1	3	0	4
Spinal misalignment	1	1	1	3
Emphysema	3	0	0	3
Congenital diaphragmatic hernia	2	0	0	2
Intrathoracic mass	1	0	0	1
Total	48	8	2	58

### Data acquisition

All patients had paediatric thoracic CT protocol (2nd generation DSCT, Siemens Somatom Definition Flash, Siemens Healthineers, Forchheim, Germany) or 16 slice MSCT (Siemens Emotion 16, Siemens Healthineers, Forchheim, Germany). All examinations were reconstructed with 1.5 mm slice thickness, increments of 1.0 mm, a dedicated lung reconstruction kernel (I70s (DSCT) or B70s (MSCT)) and soft tissue reconstruction kernel (I30s (DSCT) or B30s (MSCT)). The reconstruction kernels on the DSCT system utilized an iterative reconstruction algorithm with a strength level of 2. Image data were imported into a PACS Workstation (Aycan OsiriX PRO v.2.10, Aycan Digitalsysteme GmbH, Würzburg, Germany) and evaluated in an axial plane, as well as using multiplanar reconstructions (MPR). We used the orthogonal MPR feature in our DICOM viewer (Osirix). This allows for accurate measurements in all planes, as the data are built from 1.5 mm slices with 1.0 mm increment. The overlapping datasets ensure that the MPR images are true to the original, with no drawbacks compared to axial/coronal/sagittal reconstructions from the scanner. All CTs were reviewed and measurements recorded by one specialist in paediatric radiology.

### Measurements

**Fig. 1:**
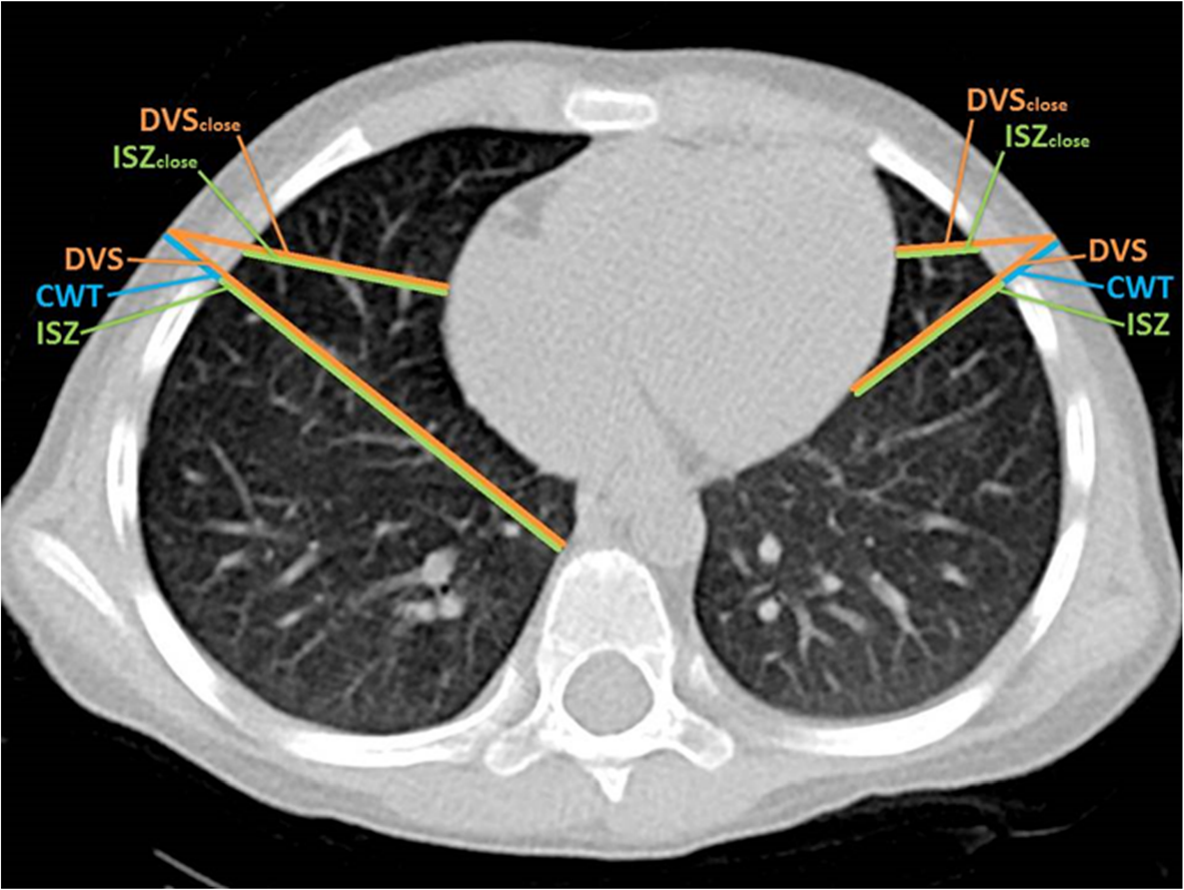
Graphical display of measurements taken. 4th ICS AAL. DVS: depth to vital structure, CWT: chest wall thickness, ISZ: intrapleural safety zone. Subscript “close” indicating measurements for a misguided puncture directed at the closest vital structure

Measurements were made at 2nd ICS MCL and 4th ICS AAL on both sides of the thorax. ICS width was measured from the inferior border of the superior rib to the superior edge of the inferior rib. Chest wall thickness and the depth to the closest vital structure were measured in various directions: in the sagittal plane at the 2nd ICS MCL (MCL_sag_), perpendicular to the chest wall at the 2nd and 4th ICS (MCL _perp_, AAL _perp_) and in a linear direction to the closest vital structure at the 2nd and 4th ICS (CWT_close_) (Fig. 1, Table 2) Intrathoracic structures classified as “vital structures” are shown in Table 3. Lung parenchyma was not classified as a vital structure. Since a pneumothorax in the prehospital setting can hardly be confirmed with a 100% accuracy and an accidental puncture of the lung parenchyma is possible, we chose to define the intraparenchymal lung vessels up to the segmental vessels as vital structures. Chest wall thickness was measured from the skin surface to the pleural cavity. Depth to vital structure was defined as the distance from the skin surface to the intersection with a vital structure. In an additional measurement, the closest vital structure to skin surface at the insertion sites was identified visually. The distance from skin surface to this reference point at the corresponding puncture site was measured. The distance for the worst case scenario; an insertion leading directly to the closest vital structure, was determined (DVS_close_). By subtracting CWT from DVS, the so called “intrapleural safety zone (ISZ)” was calculated. ISZ represents the intrathoracic distance from entering the pleural space up to the beginning of the closest vital structure (Fig. 1).

**Table 2:** Description of measurements

Measurement	Description
Width of the intercostal space	From the inferior border of the superior rib to the superior edge of the inferior rib
Chest Wall Thickness (CWT)	Skin to pleural space
Depth to vital structure (DVS)	Skin to the intersection of the insertion line (see “directions of insertion”) with an intrathoracic vital structure (see Table 3)
Intrapleural Safety Zone (ISZ)	Pleural space to the intersection with an intrathoracic vital structure (DVS – CWT), representing the intrathoracic distance to a vital structure
Directions of insertion	
Sagittal	Insertion in the sagittal plane
Perpendicular	Insertion perpendicular to the chest wall
Close	The closest vital structure to the point of insertion was identified visually. DVS was then measured from this point of reference to skin surface.

**Table 3:** Intrathoracic structures defined as “vital structures”

Pericardium	
Aorta	
SVC, IVC	
Pulmonary vessels including larger intraparenchymal branches (with the smallest easily visualized on CT-scan being the segment arteries)	
Thymus gland	

### Statistical analysis

Statistical analysis was performed using JMP 13.0 (SAS Institute Inc., Cary, NC, USA). Normally distributed data were identified using the Shapiro-Wilk W test. Continuous variables are presented as mean ± standard deviation and median and interquartile range. Comparison was made using the Mann-Whitney U test. *P*-values < 0.05 were considered statistically significant.

## Results

### Demographics

139 patients were included in the final analysis. 50 Children aged 0 years, 47 children aged 5 years and 42 children aged 10 years. Mean ages in the three groups were 0.42 (±0.32) years, 5.48 (±0.28) years and 10.45 (±0.30) years. Demographic data are shown in Table 4.

**Table 4:** Demographic data

Group	Age (mean ± SD)	Male	female	Total
0 years	0.42 (±0.32) years	29 (=58.0%)	21 (=42.0%)	50
5 years	5.48 (±0.28) years	28 (=59.6%)	19 (=40.4%)	47
10 years	10.45 (±0.30) years	28 (=66.7%)	14 (=33.3%)	42
Total	*5.16 years*	85 (=61.2%)	54 (=38.8%)	139

### 0-year-old children

The width of the ICS was significantly larger at 2nd ICS compared to 4th ICS (right: *p* < 0.05, left: *p* < 0.05). Chest wall thickness was slightly but not significantly greater at 2nd ICS than at 4th ICS. Mean required depth of puncture for successful decompression was approximately 1.4–1.6 cm at all puncture sites. DVS was significantly greater at 2nd ICS on both sides of the thorax (right: *p* < 0.05, left: *p* < 0.05), DVS_close_ however (a misguided puncture directly at the closest vital structure) was significantly smaller at 2nd ICS (right: *p* < 0.05, left: *p* < 0.05). The safe zone from penetration of the pleura to the intersection with the next vital structure (ISZ) was significantly greater for correct angles of puncture (sagittal at 2nd ICS MCL and perpendicular at 4th ICS AAL) at 2nd MCL (right: *p* < 0.05, left: *p* < 0.05). The distance to the closest vital structure in case of a deviation from recommended angle of puncture however was bigger at 4th ICS (ISZ_close_ right: *p* < 0.05, left *p* < 0.05) (Table 5).

**Table 5:** 0-year-old children, all measurements in cm ± SD, "*" indicating statistical significance

	ICS-width		CWT		CWT_close_		DVS		DVS_close_		ISZ		ISZ_close_	
	*right*	*left*	*right*	*left*	*right*	*left*	*right*	*left*	*right*	*left*	*right*	*left*	*right*	*left*
2nd ICS MCL_sag_	0.55	0.58	1.52	1.56	1.43	1.40	4.96	4.48	2.44	1.96	3.44	2.92	1.01	0.56
	[±0.18]	[±0.19]	[±0.51]	[±0.61]	[±0.41]	[±0.41]	[±1.68]	[±1.77]	[±0.63]	[±0.66]	[±1.59]	[±1.61]	[±0.61]	[±0.65]
4th ICS AAL_perp_	0.41	0.46	1.38	1.41	1.44	1.45	4.02	3.15	3.01	2.46	2.64	1.75	1.58	1.01
	[±0.13]	[±0.13]	[±0.48]	[±0.50]	[±0.48]	[±0.48]	[±1.08]	[±0.95]	[±0.78]	[±0.59]	[±0.98]	[±0.76]	[±0.70]	[±0.43]
p	< 0.05*	< 0.05*	0.11	0.15	0.94	0.72	< 0.05*	< 0.05*	< 0.05*	< 0.05*	< 0.05*	< 0.05*	< 0.05*	< 0.05*

### 5-year-old children

Width of the ICS was significantly larger at 2nd ICS (right: *p* < 0.05, left: *p* < 0.05) and mean required depth for successful puncture (CWT) was, with an average difference of 4-5 mm, significantly greater at 2nd ICS compared to 4th ICS (right: *p* < 0.05, left: *p* < 0.05). DVS was significantly larger at 2nd ICS in the left hemithorax (*p* < 0.05) but the difference did not reach statistical significance on the right (*p* = 0.14). DVS_close_ however was larger at 4th ICS on the right (*p* < 0.05) and roughly the same at 4th ICS on the left (*p* = 0.76). As a result, the ISZ was greater at 2nd ICS, but the ISZ_close_ was greater at 4th ICS (Table 6).

**Table 6:** 5-year-old children, all measurements in cm ± SD, "*" indicating statistical significance

	ICS-width		CWT		CWT_close_		DVS		DVS_close_		ISZ		ISZ_close_	
	*right*	*left*	*right*	*left*	*right*	*left*	*right*	*left*	*right*	*left*	*right*	*left*	*right*	*left*
2nd ICS MCL_sag_	1.35	1.43	1.76	1.81	1.69	1.69	6.90	6.78	3.53	3.10	5.14	4.97	1.83	1.42
	[±0.31]	[±0.36]	[±0.48]	[±0.48]	[±0.46]	[±0.48]	[±2.43]	[±2.67]	[±0.70]	[±0.91]	[±2.46]	[±2.68]	[±0.77]	[±1.03]
4th ICS AAL_perp_	0.72	0.83	1.34	1.28	1.42	1.37	5.98	4.29	4.01	3.00	4.64	3.00	2.58	1.63
	[±0.18]	[±0.23]	[±0.46]	[±0.41]	[±0.50]	[±0.44]	[±1.60]	[±1.29]	[±1.00]	[±0.70]	[±1.72]	[±1.33]	[±1.01]	[±0.66]
p	< 0.05*	< 0.05*	< 0.05*	< 0.05*	< 0.05*	< 0.05*	0.14	< 0.05*	< 0.05*	0.76	0.82	< 0.05*	< 0.05*	0.16

### 10-year-old children

Width of the ICS was significantly greater at 2nd ICS (right: *p* < 0.05, left: *p* < 0.05) and mean required depth for successful puncture (CWT) was significantly greater at 2nd ICS (2.6 cm at 2nd ICS and 2.2 cm at 4th ICS, right: *p* < 0.05, left: *p* < 0.05)). DVS was greater at 2nd MCL on both sides for correct angle of puncture but did only reach statistical significance on the left side (right: *p* = 0.13, left: *p* < 0.05). On the left hemithorax DVS_close_ was nearly the same at 2nd and 4th ICS (*p* = 0.93), and slightly smaller than on the right side (*p* = 0.11). The ISZ was bigger at 2nd ICS (right: *p* = 0.58, left *p* < 0.05), but ISZ_close_ was bigger at 4th ICS (right: *p* < 0.05, left: *p* = 0.26) (Table 7).

**Table 7:** 10-year-old children, all measurements in cm ± SD, "*" indicating statistical significance

	ICS-width		CWT		CWT_close_		DVS		DVS_close_		ISZ		ISZ_close_	
	*right*	*left*	*right*	*left*	*right*	*left*	*right*	*left*	*right*	*left*	*right*	*left*	*right*	*left*
2nd ICS MCL_sag_	1.58	1.67	2.61	2.63	2.61	2.59	9.20	9.18	4.94	4.33	6.59	6.55	2.33	1.75
	[±0.31]	[±0.34]	[±1.15]	[±1.23]	[±1.17]	[±1.28]	[±2.94]	[±3.36]	[±1.17]	[±1.43]	[±2.98]	[±3.29]	[±1.08]	[±1.29]
4th ICS AAL_perp_	1.07	1.16	2.21	2.19	2.32	2.32	8.00	6.11	5.52	4.36	5.79	3.92	3.19	2.04
	[±0.33]	[±0.37]	[±1.33]	[±1.30]	[±1.34]	[±1.39]	[±2.29]	[±1.81]	[±1.76]	[±1.44]	[±2.12]	[±1.53]	[±1.36]	[±0.95]
p	< 0.05*	< 0.05*	< 0.05*	< 0.05*	0.07	0.11	0.13	< 0.05*	0.11	0.93	0.58	< 0.05*	< 0.05*	0.26

**Fig. 2:**
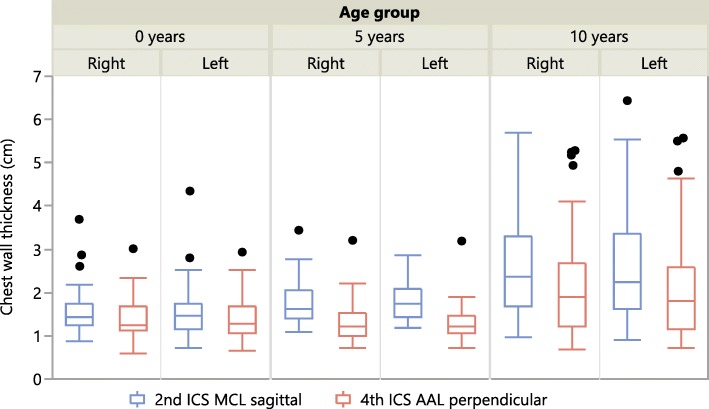
Chest Wall Thickness. Median, 1st and 3rd Quartile, Minimum, Maximum and Outliers

The results for CWT in all age groups are presented graphically in Fig. 2.

### Structures directly adjacent to the thoracic wall

**Fig. 3:**
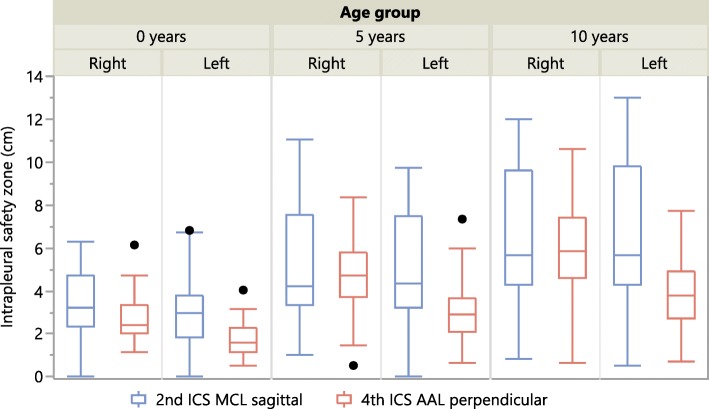
Intrapleural Safety Zone. Median, 1st and 3rd Quartile, Minimum, Maximum and Outliers

Figure 3 shows the results for “intrapleural safety zone” for the commonly recommended puncture techniques (sagittal puncture at the 2nd ICS MCL and perpendicular puncture at the 4th ICS AAL). As can be seen from the whiskers in the diagram, the safety zone was remarkably small or even zero for some outliers. In the group of infants, the thymus gland was found directly adjacent to the thoracic wall in two infants in the right hemithorax and one infant in the left hemithorax at 2nd ICS MCL. The closest vital structure at this insertion site was the heart, which was found lying only slightly medially to the 2nd ICS MCL on the left. With a puncture directed more medially (at the closest vital structure, MCL_close)_ the heart was found adjacent to the chest wall in 8/50 (16%) patients on the left and would have been punctured immediately after penetration of the pleura. At 4th ICS AAL, regardless of direction of puncture, no vital structures were found directly adjacent to the chest wall. In one 5-year-old child the heart was found adjacent to the chest wall at 2nd ICS MCL for sagittal puncture on the left. Even a slight deviation (puncture not in the sagittal plane but perpendicular to the chest wall) resulted in the heart being adjacent to the thoracic wall in another child. At 4th ICS AAL no vital structures were found adjacent to the thoracic wall along the path of the needle. In the 10-year-old children, no vital structures were found adjacent to the thoracic wall at 2nd ICS MCL and 4th ICS AAL. Detailed results are shown in the (Additional file 1: Table S1–S3).

## Discussion

Trauma guidelines traditionally recommended 2nd ICS MCL for needle decompression of tension pneumothorax [1, 2]. Lately, the 4th and 5th ICS AAL and MAL (midaxillary line) have been recommended as sites of choice by ATLS (Advanced Trauma Life Support) and TCCC (Tactical Combat Casualty Care) guidelines for adult patients [3, 4]. No specific recommendations are made for children. There are no widely accepted published guidelines on insertion depth in children. Thoracic trauma however, affects around 20% of moderately to severely injured children [10] and up to 50% of critically injured children [11]. In an analysis from the German Trauma Registry regarding severe Injuries (AIS ≥ 3), chest trauma was the second most common injury in infants, toddlers and pre-schoolers [12]. Ismail et al. report an incidence of in-hospital need for pleural decompression in paediatric patients with chest trauma of 24.2% [13]. Tension pneumothorax in children can become rapidly fatal, like in adults. Decompression therefore is often a procedure that has to be performed with high urgency to avoid potentially preventable cardiac arrest. In a cohort of adult traumatic cardiac arrest patients, Kleber et al. report missing or insufficient chest decompression in 37% of the patients with tension pneumothorax [14]. In a retrospective analysis of children injured during the Afghanistan war, Sokol et al. found that only 14/95 patients with an indication for pleural decompression received a prehospital intervention [15]. Especially in children, experience of Emergency Medicine Service (EMS) personnel performing needle decompression is scarce, let alone open finger thoracostomy. Carlson et al. report an incidence of prehospital pleural decompression in children of only 0.3 per 1000 paediatric EMS responses [16]. This may be due to underdiagnosis, unwillingness to intervene or rarity of the pathology. We therefore conducted this CT-based study to provide guidance regarding the optimal and safest required depth and location of puncture for successful decompression of tension pneumothorax and evaluate the accompanying risks at different puncture sites in three different age groups (0, 5, 10 years).

The width of the intercostal space was significantly lower at 4th ICS AAL in all age groups. The very small width of the ICS in infants (Mean: 4.1–5.8 mm at the investigated puncture sites, Table 5) poses a significant risk of injuring the intercostal vessels when using large bore cannulas inserted at the incorrect site (intercostal neuromuscular bundle). Laceration of the intercostal artery with subsequent need for surgical intervention as a complication of needle thoracostomy has been described in several case reports for adult patients and has to be taken into account when choosing the cannula bore for decompression [17]. The recommendation of using the cannula with the maximum diameter possible might lead to a serious risk of injury especially in small children [18]. A compromise between better decompression by the higher flow rates of large bore cannulas and the risk of intercostal vessel laceration has to be found. Moreover, the technique of simple open thoracostomy, which is recommended in adults [2] and was recently recommended for children as a result of a Delphi process in the United Kingdom and Ireland [19], is technically not easy for the prehospital or in-hospital provider due to the small intercostal diameters. Decompression failure due to CWT exceeding needle catheter length is a commonly reported phenomenon in adults [6]. In our study mean CWT ranged from 1.56–1.80 cm in 0-year-old children, 1.28–1.81 cm in 5-year-old children and 2.19–2.63 cm in 10-year-old children. CWT was about 10–25% higher at 2nd ICS MCL_sag_ compared to 4th ICS AAL_perp_. Mandt et al. just recently reported on appropriate needle length for pleural decompression in paediatric patients [20]. The authors measured chest wall thickness in computed tomography scans at 2nd ICS MCL and 4th ICS AAL in four age groups based on Broselow™ colour. In the main, the reported results are congruent with the measurements in our study. The CWT reported by Mandt el al however is slightly larger than in our study, but the groups used by the authors do not exactly match our age groups, which hinders a direct and exact comparison of the results. Nevertheless, the reported differences are within a range of a few millimetres and do not have to be considered as clinically relevant. Using a 4.5 cm catheter, decompression would have been successful at all puncture sites in the 0 and 5-year-old children and in around 90% of the 10-year-old patients. The use of longer and larger bore catheters however increases the risk of injury to the intercostal vessels and intrathoracic structures. The evaluation of different needle types in the investigated age groups, puncture sites and the most favourable ratio of successful decompression to injury risk is part of another study by our group.

### Risk of injury to vital structures

In presence of a pneumothorax, the air entrapped in the pleural cavity provides a buffer zone for needle puncture, keeping the lung and most likely vital structures away from the chest wall. Clinical diagnosis of a pneumothorax however can be challenging and is likely over-diagnosed especially in the prehospital setting [21]. Thoracic ultrasound is an option to optimize the diagnosis of a pneumothorax but is not universally available prehospital. Nevertheless, false positive diagnosis due to tracheal tube displacement, diaphragmatic rupture, pulmonary contusion or ventilation disturbances is reported in up to 4.5% [22]. Eckstein et al. report a rate of iatrogenic pneumothorax caused by needle decompression without indication of 2% [23]. Due to publication bias the true rate is most likely clearly higher. In their aforementioned work, Sokol et al. report of 16 prehospital pleural decompressions in children, of which 2 (12.5%) were performed without a clear indication [15]. These data show that a false positive diagnosis of tension pneumothorax has to be considered and the risk of injury to intrathoracic vital structures has to be taken into account when choosing the site for needle decompression. So far, to the best of our knowledge, no specific data for children regarding depth to vital intrathoracic organs exist. The risk of injury to vital intrathoracic organs was therefore assessed in further detail in this study.

### Risk of injury in infants

When comparing the two possible puncture sites (2nd ICS MCL_sag_ and 4th ICS AAL_perp_) in infants, CWT was roughly the same and DVS was greater at 2nd MCL_sag_. As a result, the ISZ was greatest for 2nd ICS MCL_sag_ on both sides of the thorax. The closest vital structure however was found in closer proximity at 2nd ICS MCL. Furthermore, Fig. 3 shows that vital structures (heart, thymus gland) were found directly adjacent to the thoracic wall in several infants. In absence of a pneumothorax the thymus gland would have been hit at the 2nd ICS MCL in two infants on the right and one infant on the left side, even with a correctly directed needle in the sagittal plane. With a misguided puncture, on the left hemithorax, the heart could have been hit immediately after penetration of the thoracic wall in 16% of the infants. From our point of view, in infants 2nd ICS MCL should only be used after definitive point of care ultrasound/radiographic confirmation of a pneumothorax and the puncture should strictly be performed in the sagittal plane. At 4th ICS AAL no vital structures were found directly adjacent to the chest wall in any direction of puncture in this age group. However, the narrow ICS and the smaller ISZ (DVS – CWT) should be kept in mind at this site of puncture.

### Risk of injury to vital structures in 5-year-old children

CWT was smaller at 4th ICS AAL and DVS was greatest for 2nd ICS MCL_sag_. In this age group, the heart was found adjacent to the thoracic wall in one child for sagittal puncture and two children for misguided puncture directed at the closest vital structure at 2nd ICS MCL. Therefore, the risk of puncturing the heart is smaller than in infants, but still present. At 4th ICS AAL no vital structures were directly adjacent, but, as in infants, ISZ (DVS – CWT) was smaller.

### Risk of injury to vital structures in 10-year-old children

For correct direction of puncture (2nd ICS MCL_sag_ or 4th ICS AAL_perp_) no vital structures were directly adjacent to the chest wall. For incorrect angle of needle entry however, the heart could have been injured directly in 16.7% of the patients at 2nd ICS MCL and 2.4% at 4th ICS AAL.

The measures DVS and ISZ suggest a bigger “safety zone” at 2nd ICS in all age groups. Any deviation from correct angle of entry towards the closest vital structure however leads to the opposite result, with DVS_close_ and ISZ_close_ being greater at 4th ICS. The higher cardiothoracic ratio and, especially in expiration, the more transverse position of the heart in infants, toddlers and pre-school children leads to a closer proximity of the left ventricle to the 2nd ICS MCL on the left hemithorax. This phenomenon is regularly observed in paediatric point-of-care ultrasound examinations of the chest. In summary it seems there is no benefit but increased risk of harm in choosing the 2nd ICS MCL as insertion site.

### Limitations

This study has several limitations. First of all, measurements were taken in children without pneumothorax. Presence of pneumothorax would minimize the risk of injury to vital structures when puncture is performed under aspiration via syringe and stopped immediately after aspiration of air. However, needle insertion by landmark is not accurate and tension pneumothorax is probably over diagnosed outside the context of POCUS/radiography. Hence our study findings are relevant. Secondly, the extent of compression of the subcutaneous tissue by the needle tip cannot be measured in CT reliably. Compression might lead to reduced CWT and therefore DVS especially in obese children. Thirdly, we were not able to collect data on the height and weight of the children, which might offer a better correlation with CWT than age [24]. All measurements were recorded by one single investigator. Reproducibility of the measurements was therefore not assessed. Furthermore, we did not record the angle of entry for puncture directed at the closest vital structure. The degree of deviation from the recommended angle that would lead to injury can therefore not be specificied but deviation that would cause injury can be seen. Finally, we identified puncture sites on CT. Ferrie et al. showed a low accuracy among emergency physicians in identifying correct landmarks for needle thoracocentesis in adults with a trend to perform punctures medial to the MCL [25]. In an adult cadaveric study, Inaba et al. found a significantly higher rate of correct needle placement for the 5th ICS compared to the 2nd ICS [26, 27]. In the paediatric population, where bony landmarks are less obvious and smaller spatial relationships are present, there may be increased risk of error in placement position with potential significant iatrogenic injury (e.g. heart) but this was not directly evaluated in our study.

## Conclusion

Based on this study we recommend the 4th ICS AAL as the primary site for needle decompression in tension pneumothorax. As the heart and thymus gland were found directly adjacent to the thoracic wall at 2nd ICS MCL in several children aged 0 and 5 years, this puncture site cannot be recommended unless a pneumothorax in this region is confirmed. The caveat is that although the 4th ICS AAL offers a smaller chest wall thickness, the width of the ICS is narrower and hence the risk of neurovascular bundle injury is slightly increased. However, the difference in width compared to 2nd ICS may not be clinically significant in terms of needle insertion. Deviations from correct angle of entry at 2nd ICS however are accompanied by higher risk of injury than at 4th ICS. To avoid an unnecessarily deep needle penetration, puncture should be performed under guidance by aspiration of air via a syringe and needle movement should be immediately stopped after aspiration of air. Whenever possible ultrasound should be used for confirmation of a pneumothorax, to measure chest wall thickness and confirm lack of underlying vital structure (e.g. heart) before puncture and hence minimize depth of needle insertion and reduce the risk of injuring vital structures. Whenever ultrasound is not available, knowledge on depth to vital structures as well as knowledge concerning chest wall thickness is essential to avoid serious injuries in children. Depth markers on the needle would be helpful for judging depth of needle penetration. Furthermore, a small skin incision prior to puncture can reduce the force needed for advancement of the needle and therefore offer a better control of the depth of puncture. An age appropriate device with a Veress tip could also be an alternative to reduce complications associated with needle decompression.

## Additional file

Additional file 1: **Table S1.** Structures directly adjacent to the thoracic wall, 0-year-old children. **Table S2.** Structures directly adjacent to the thoracic wall, 5-year-old children. **Table S3.** Structures directly adjacent to the thoracic wall, 10-year-old children. (DOC 67 kb)

## Abbreviations

AAL: Anterior axillary line
CWT: Chest wall thickness
DVS: Depth to vital structure
DVS_perp_: Depth to vital structure, direction of puncture perpendicular to the chest wall
ICS: Intercostal space
MCL: Medioclavicular line
SD: Standard deviation
DVS_sag_: Depth to vital structure, direction of puncture in the sagittal plane
DVS_close_: Depth to vital structure, puncture straight in the direction of the closest vital structure
ISZ: Intrapleural safety zone
ISZ_close_: Intrapleural safety zone, puncture straight in the direction of the closest vital
CT: Computed tomography
EMS: Emergency medicine service
ATLS: Advanced trauma life support
TCCC: Tactical combat casualty care
