# Supplementary material for: Chest wall thickness and depth to vital structures in paediatric patients – implications for prehospital needle decompression of tension pneumothorax
Source: Scand J Trauma Resusc Emerg Med. 2019 Apr 16;27:45. doi: 10.1186/s13049-019-0623-5 (PMC6469218; doi:10.1186/s13049-019-0623-5)
Supplement: Supplementary file 1 — Table S1. Structures directly adjacent to the thoracic wall, 0-year-old children. Table S2. Structures directly adjacent to the thoracic wall, 5-year-old children. Table S3. Structures directly adjacent to the thoracic wall, 10-year-old children. (DOC 67 kb) [file 13049_2019_623_MOESM1_ESM.doc]

**Additional file 1**

**Table S1:** Structures directly adjacent to the thoracic wall, 0-year-old children

| **Right hemithorax** | **2nd ICS** | | | **4th ICS** | |
| --- | --- | --- | --- | --- | --- |
|  | **MCLperp** | **MCLsag** | **MCLclose** | **AALperp** | **AALclose** |
| None | 48 (96%) | 48 (96%) | 46 (92%) | 50 (100%) | 50 (100%) |
| Heart | 0 (0%) | 0 (0%) | 0 (0%) | 0 (0%) | 0 (0%) |
| Thymus gland | 2 (4%) | 2 (4%) | 4 (8%) | 0 (0%) | 0 (0%) |
|  |  |  |  |  |  |
| **Left hemithorax** | **2nd ICS** | | | **4th ICS** | |
|  | **MCLperp** | **MCLsag** | **MCLclose** | **AALperp** | **AALclose** |
| None | 46 (92%) | 49 (98%) | 31 (62%) | 50 (100%) | 50 (100%) |
| Heart | 1 (2%) | 0 (0%) | 8 (16%) | 0 (0%) | 0 (0%) |
| Thymus gland | 3 (6%) | 1 (2%) | 11 (22%) | 0 (0%) | 0 (0%) |

**Table S2:** Structures directly adjacent to the thoracic wall, 5-year-old children

| **Right hemithorax** | **2nd ICS** | | | **4th ICS** | |
| --- | --- | --- | --- | --- | --- |
|  | **MCLperp** | **MCLsag** | **MCLclose** | **AALperp** | **AALclose** |
| None | 47 (100%) | 47 (100%) | 47 (100%) | 47 (100%) | 47 (100%) |
| Heart | 0 (0%) | 0 (0%) | 0 (0%) | 0 (0%) | 0 (0%) |
| Thymus gland | 0 (0%) | 0 (0%) | 0 (0%) | 0 (0%) | 0 (0%) |
|  |  |  |  |  |  |
| **Left hemithorax** | **2nd ICS** | | | **4th ICS** | |
|  | **MCLperp** | **MCLsag** | **MCLclose** | **AALperp** | **AALclose** |
| None | 45 (95,7%) | 46 (97,9%) | 45 (95,7%) | 47 (100%) | 47 (100%) |
| Heart | 2 (4,3%) | 1 (2,1)% | 2 (4,3%) | 0 (0%) | 0 (0%) |
| Thymus gland | 0 (0%) | 0 (0%) | 0 (0%) | 0 (0%) | 0 (0%) |

**Table S3:** Structures directly adjacent to the thoracic wall, 10-year-old children

| **Right hemithorax** | **2nd ICS** | | | **4th ICS** | |
| --- | --- | --- | --- | --- | --- |
|  | **MCLperp** | **MCLsag** | **MCLclose** | **AALperp** | **AALclose** |
| None | 42 (100%) | 42 (100%) | 42 (100%) | 42 (100%) | 42 (100%) |
| Heart | 0 (0%) | 0 (0%) | 0 (0%) | 0 (0%) | 0 (0%) |
| Thymus gland | 0 (0%) | 0 (0%) | 0 (0%) | 0 (0%) | 0 (0%) |
|  |  |  |  |  |  |
| **Left hemithorax** | **2nd ICS** | | | **4th ICS** | |
|  | **MCLperp** | **MCLsag** | **MCLclose** | **AALperp** | **AALclose** |
| None | 42 (100%) | 42 (100%) | 35 (83,3%) | 42 (100%) | 41 (97,6%) |
| Heart | 0 (0%) | 0 (0%) | 7 (16,7%) | 0 (0%) | 1 (2,4%) |
| Thymus gland | 0 (0%) | 0 (0%) | 0 (0%) | 0 (0%) | 0 (0%) |
